# Rare finding of concomitant pseudomelanosis of stomach and duodenum; case report and literature review 

**Published:** 2018

**Authors:** Tagore Sunkara, Megan E Caughey, Vinaya Gaduputi

**Affiliations:** 1 *Division of Gastroenterology and Hepatology, The Brooklyn Hospital Center, Clinical Affiliate of The Mount Sinai Hospital, 121 Dekalb Ave, Brooklyn, New York, USA*; 2 *New York Institute of Technology College of Osteopathic Medicine, Old Westbury, New York, USA*; 3 *Division of Gastroenterology and Hepatology, SBH Health System, 4422 Third Ave, Bronx, New York, USA*

**Keywords:** Pseudomelanosis, Melanosis gastri, Melanosis duodeni, Hyperpigmentation

## Abstract

Melanosis of the stomach and duodenum is a rare entity and a striking finding diagnosed by upper gastrointestinal endoscopy. Here, we describe the case of an 83-year-old female, with a complicated medical history, who was referred to gastroenterologist to assess bleeding risk. From the endoscopy, it was determined that she had both melanosis gastri and duodeni. Although both are rare, gastric melanosis appears to be even more unusual than duodenal melanosis, with only a few reported cases documented in the literature thus far.

## Introduction

 Melanosis of the stomach and duodenum is a rare entity and a striking finding diagnosed by upper gastrointestinal endoscopy. Here, we describe the case of an 83-year-old female, with a complicated medical history, who was referred to gastroenterologist to assess bleeding risk. From the endoscopy, it was determined that she had both melanosis gastri and duodeni. Although both are rare, gastric melanosis appears to be even more unusual than duodenal melanosis, with only a few reported cases documented in the literature thus far. 

## Case Report

An 83-year-old female was referred to gastroenterologist by a cardiologist for a determination of her bleeding risk in the context of possible anticoagulation for atrial fibrillation. Her past medical history includedatrial fibrillation (not on anticoagulation or aspirin due to prior history of a gastrointestinal (GI) bleed), chronic obstructive pulmonary disease, pulmonary hypertension, chronic heart failure, and non-Hodgkin's lymphoma treated with chemotherapy twenty-two years earlier. The patient had been taking Xarelto for atrial fibrillation, but following an episode of gastrointestinal bleeding two years prior, the patient’s Xarelto was discontinued. 

**Figure 1:**
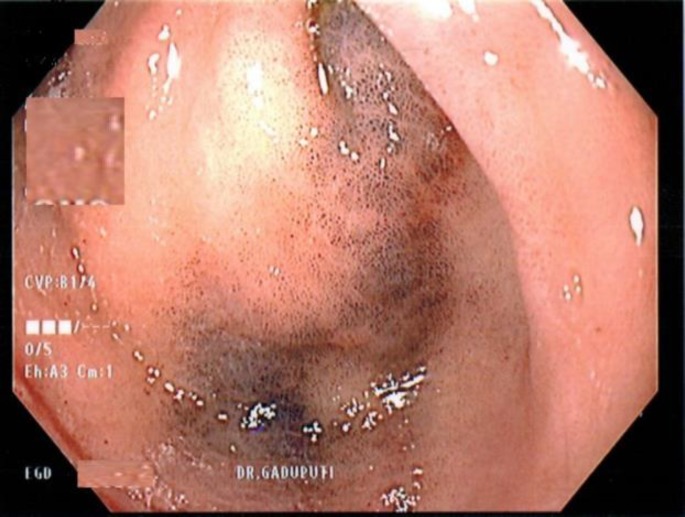
Endoscopy showing mucosal changes in the stomach (antrum) that resembled melanosis gastri.

**Figure 2:**
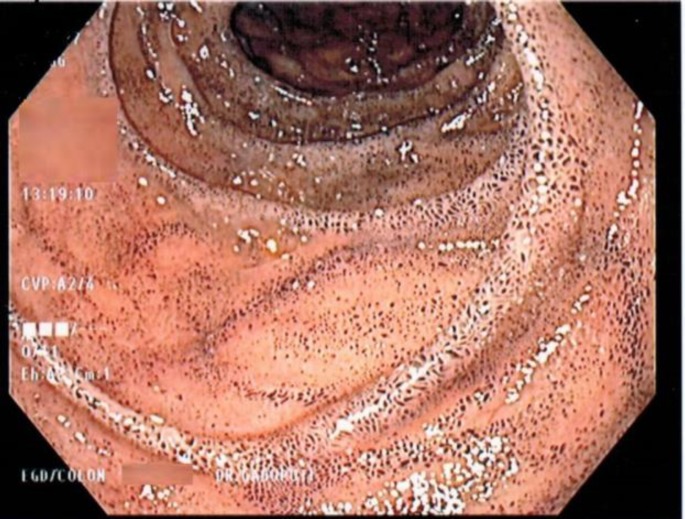
Endoscopy showing mucosal changes in the duodenum that resembled melanosis duodeni

A cardiologist had recently evaluated the patient for atrial fibrillation and consulted gastroenterologist to determine the patient’s risk for GI bleeding before restarting anticoagulation therapy. The patient was noted to have normocytic, normochromic anemia with hemoglobin of 10 gm/dL and mean corpuscular volume of 82; however, additional findings were not consistent with iron deficiency anemia. As a result, the patient received a diagnostic endoscopy and colonoscopy to evaluate for any high-risk lesions. The endoscopy discovered patchy erythematous antral gastropathy and mucosal changes in the stomach and the duodenum that resembled melanosis gastri (Figure 1) and duodeni (Figure 2). Pathology report of this stomach and duodenal biopsy ultimately determined that the specimen had normal villous architecture without diagnostic abnormality. 

The colonoscopy also revealed severe diverticulosis and luminal distortion, to the extent that the scope could not be advanced safely beyond the distal descending colon, as well as non-bleeding small internal hemorrhoids. The patient was instructed to avoid NSAIDS and consume a high-fiber diet. Upon follow-up visit, she had no complaints but decided against receiving anticoagulation therapy due to a risk of bleeding. 

## Discussion

A benign finding of gastric and duodenal melanosis are such infrequent and manifest conditions that can lead to an extensive, expensive, and ultimately unnecessary work-up. It involves the collection of dark pigmented granules inside the macrophages of the duodenal villi’s lamina propria, usually within the apical tips (2). Prussian blue and Fontana-Masson stains can be used to detect these pigments in macrophages located within the lamina propria (3).

The dark, spotted appearance of the mucosa is predominately due to the sequestration of ferrous sulfate in the macrophages, which explains why those taking iron supplements might be at greater risk of developing melanosis. Two potential mechanisms have emerged to explain the pathogenesis of gastrointestinal melanosis. One theory is that melanosis results from compromised luminal iron transport within the context of oral iron supplementation. Alternatively, melanosis could be a consequence of mucosal iron deposition following intramucosal hemorrhage. Electron probe microanalysis though has revealed that these same macrophages also contain hemosiderin, lipofuscin, and lipomelanin (4).

Gastric and duodenal melanosis represents a diagnostic challenge for physicians; however, the precise etiology and clinical significance of this condition remain unknown. It is typically seen in older women and has been associated with several clinical conditions, some of which include: hypertension, hemochromatosis, diabetes, chronic renal disease, and gastrointestinal bleeding. Gastric and duodenal melanosis has also been seen in connection with medications containing ferrous sulfate as well as with antihypertensive medications, like furosemide, hydrochlorothiazide, propranolol, and hydralazine (5).

More recently, it has been proposed that there may be a correlation between the pigmentation of melanosis gastri or duodeni and impaired absorption of nutrients in these areas. When absorption is hindered, dyspepsia can result, which speaks why so many patients with melanosis present with discomfort and indigestion first. However, many of those diagnosed with melanosis gastri or duodeni remain entirely asymptomatic.

Additionally, one study examined colon biopsies of patients prescribed anthraquinone laxatives for an extended period of time. This study determined that although only fourteen patients were identified as having discoloration on colonoscopy, a total of forty-five patients were diagnosed histologically with melanosis coli. Although this study examined the colon and not the small bowel, this is a particularly interesting finding because most of these patients suffered from constipation, abdominal pains, and distention. From radiographic studies, these same patients were found to have motility disorders and abnormal absorptive epithelial cells were identified on electron microscopy in addition to degenerated nerve fibers (6). Overall, the exact etiology or pathophysiology of gastric and duodenal melanosis remains a mystery. Studies like the previous one suggest that the melanosis may be related to atypical absorptive processes. Ultimately though, more researches are needed to develop a specific protocol for the treatment and follow-up of this extremely rare condition, taking into account the spectrum of symptom severity. 

Although melanosis proves a rarity, there have been a few documented instances of it. One case report described an 88-year-old female patient with a past medical history of essential hypertension, chronic renal failure, and diabetes mellitus, who complained of abdominal pain. In addition to these comorbidities associated with melanosis, the patient was prescribed furosemide and iron supplements long-term. An upper GI endoscopy revealed gastric and duodenal discoloration, and a biopsy confirmed the diagnosis of melanosis gastri and duodeni (5).

Similarly, in another case report, a 70-year-old woman, with iron deficiency and chronic kidney disease also complained of abdominal pain for two weeks. An upper endoscopy identified black, speckled gastric and duodenal mucosa, and biopsy specimens stained positive for the Fontana-Masson trichrome stain, officially diagnosing the patient with melanosis. Both of the patients in these two case reports were elderly women with some of the same comorbidities (7).

A case series of four female patients and one male patient, who were on average 70-years-old, determined that all four patients had duodenal pseudomelanosis, whereas only one patient had gastric antral pseudomelanosis. These patients exhibited some of the following medical conditions: iron deficiency anemia, hypertension, chronic kidney disease, hydralazine use, and iron supplementation. Diagnoses of pseudomelanosis in these individuals were made based on biopsy specimens staining positive for iron but negative for calcium and copper (8).

Lastly, in addition to these isolated case reports and this case series, there are two additional documented instances of patients diagnosed with pseudomelanosis of the stomach, duodenum, and jejunum. These patients were 60-years-old and 74-years-old, which is consistent with the ages of the other patients who received this same diagnosis. The 60-year-old patient presented with a milder anemia and hematemesis but was found to have the distinctive gray-black pigmentation pattern on endoscopy (9). The 74-year-old patient presented with anemia 4 years earlier, for which she had been taking ferrous sulfate (3 times a day) in addition to hydralazine and furosemide for chronic renal insufficiency, all likely contributing to the pseudomelanosis (10). Thus, the extensive literature review we performed suggests that gastric and duodenal melanosis is a truly unusual condition. Those diagnosed with melanosis share a similar profile of characteristics, risk factors, and comorbidities, but the diagnosis itself remains one of indeterminate clinical consequence that warrants further investigation.

## Conflict of interests

The authors declare that they have no conflict of interest.
